# Morpho-Phylo Taxonomy of Novel Dothideomycetous Fungi Associated With Dead Woody Twigs in Yunnan Province, China

**DOI:** 10.3389/fmicb.2021.654683

**Published:** 2021-03-23

**Authors:** Peter E. Mortimer, Rajesh Jeewon, Jian-Chu Xu, Saisamorn Lumyong, Dhanushka N. Wanasinghe

**Affiliations:** ^1^CAS Key Laboratory for Plant Biodiversity and Biogeography of East Asia (KLPB), Kunming Institute of Botany, Chinese Academy of Sciences, Kunming, China; ^2^Department of Biology, Faculty of Science, Chiang Mai University, Chiang Mai, Thailand; ^3^Honghe Center for Mountain Futures, Kunming Institute of Botany, Yunnan, China; ^4^Department of Health Sciences, Faculty of Medicine and Health Sciences, University of Mauritius, Reduit, Mauritius; ^5^World Agroforestry Centre, East and Central Asia, Kunming, China; ^6^Faculty of Science, Research Center of Microbial Diversity and Sustainable Utilization, Chiang Mai University, Chiang Mai, Thailand; ^7^Academy of Science, The Royal Society of Thailand, Bangkok, Thailand

**Keywords:** Ascomycota, coelomycetes, Honghe, Kunming, Pleosporales, Yuxi

## Abstract

Within the field of mycology, macrofungi have been relatively well-studied when compared to microfungi. However, the diversity and distribution of microfungi inhabiting woody material have not received the same degree of research attention, especially in relatively unexplored regions, such as Yunnan Province, China. To help address this knowledge gap, we collected and examined fungal specimens from different plants at various locations across Yunnan Province. Our investigation led to the discovery of four species that are clearly distinct from extant ones. These taxonomic novelties were recognized based on morphological comparisons coupled with phylogenetic analyses of multiple gene sequences (non-translated loci and protein-coding regions). The monotypic genus *Neoheleiosa* gen. nov. (type: *N*. *lincangensis*) is introduced in Monoblastiaceae (Monoblastiales) for a woody-based saprobic ascomycete that possesses globose to subglobose or obpyriform ascomata with centric or eccentric, papillate ostioles, an ascomatal wall with thin-walled cells of textura globulosa, cylindric, pedicellate asci with an ocular chamber, and 1-septate, brown, guttulate, longitudinally striated, bicellular ascospores. *Neoheleiosa* has a close phylogenetic affinity to *Heleiosa*, nevertheless, it is morphologically dissimilar by its peridium cells and ornamented ascospores. *Acrocalymma hongheense* and *A*. *yuxiense* are described and illustrated as new species in Acrocalymmaceae. *Acrocalymma hongheense* is introduced with sexual and asexual (coelomycetous) features. The sexual morph is characterized by globose to subglobose, ostiolate ascomata, a peridium with textura angularis cells, cylindric-clavate asci with a furcate to truncate pedicel and an ocular chamber, hyaline, fusiform, 1-septate ascospores which are surrounded by a thick, distinct sheath, and the asexual morph is featured by pycnidial conidiomata, subcylindrical, hyaline, smooth, annelledic, conidiogenous cells, hyaline, guttulate, subcylindrical, aseptate conidia with mucoid ooze at the apex and with a rounded hilum at the base. *Acrocalymma yuxiense* is phylogenetically distinct from other extant species of *Acrocalymma* and differs from other taxa in *Acrocalymma* in having conidia with three vertical eusepta. *Magnibotryascoma kunmingense* sp. nov. is accommodated in Teichosporaceae based on its coelomycetous asexual morph which is characterized by pycnidial, globose to subglobose, papillate conidiomata, enteroblastic, annelledic, discrete, cylindrical to oblong, hyaline conidiogenous cells arising from the inner layer of pycnidium wall, subglobose, oval, guttulate, pale brown and unicelled conidia.

## Introduction

Dothideomycetes is the largest and most ecologically diverse class of Ascomycota (Hongsanan et al., 2020a), consisting of 28,729 species (Kirk, 2019). This class comprises saprobes, human and plant pathogens, endophytes, epiphytes, lichens, lichenicolous, nematode-trapping and rock-inhabiting members (Jeewon et al., 2017, 2018; Zhang J.F. et al., 2019; Hongsanan et al., 2020a). Hyde et al. (2013) provided the first comprehensive monograph of the families in Dothideomycetes. Since then, the taxonomies of Dothideomycetes have been updated with new taxa in several journal series, e.g., *Fungal Diversity notes*, *Fungal planet description sheets*, *Mycosphere notes*, *Fungal Biodiversity Profiles*, *Fungal Systematics and Evolution*, *New and Interesting Fungi*. Wijayawardene et al. (2014) provided an outline for the proposals of protection or suppression of generic names of Dothideomycetes. Consistent with the “one fungus-one name” concept, Rossman et al. (2015) provided recommendations for the nomenclature of pleomorphic genera in the class. Attributable to the continual changes of the taxa in this class, the taxonomy of Dothideomycetes is in a perpetual transitional state (Pem et al., 2019a), and as such, the outline of this class has been frequently revised (Wijayawardene et al., 2017, 2020). Recent publications by Hongsanan et al. (2020a, b) expanded the taxonomic concepts of families in the Dothideomycetidae, Pleosporomycetidae, and orders and families incertae sedis in Dothideomycetes. Hongsanan et al. (2020a, b) have accepted 38 orders and 210 families in Dothideomycetes. In order to provide a suitable platform to bring these data together, the website https://www.dothideomycetes.org was established by Pem et al. (2019a). Liu et al. (2017) proposed divergence time estimates as additional evidence for rearranging the internal classification of this class and this has been helpful to establish new families and species (Zhang S.N. et al., 2019, Bhunjun et al., 2021). The most recent order-level multi-gene phylogeny for Dothideomycetes is provided by Maharachchikumbura et al. (2021), which also introduced another two orders *viz*. Homortomycetales and Holmiellales, bringing the total number of orders to 40 in the class.

Monoblastiaceae is the only family in Monoblastiales comprising both lichenized and non-lichenized ascomycetes. Wijayawardene et al. (2020) accepted six genera in this family. Initiated by Hyde et al. (2020); Hongsanan et al. (2020b) synonymized Eriomycetaceae under Monoblastiaceae. Accordingly, this family currently includes 11 genera (Hongsanan et al., 2020b). The majority of these fungi grow on bark in tropical forests, but the family is also commonly found in leaf-inhabiting lichen communities (Aptroot and Sipman, 1993; Lücking, 2008). These foliicolous lichens can be useful in monitoring the environmental health of tropical forest ecosystems (Hongsanan et al., 2020b).

Alcorn and Irwin (1987) introduced *Acrocalymma* to accommodate *A*. *medicaginis*, which was previously identified as *Stagonospora meliloti*, known for causing root and foliar rot of *Medicago sativa* in Australia. In the phylogenetic analyses of Trakunyingcharoen et al. (2014), *Acrocalymma* species (*A*. *aquatica*, *A*. *cycadis*, *A*. *fici*, *A*. *medicaginis*, and *A*. *vagum*) represented an undefined lineage in Dothideomycetes for which the family name Acrocalymmaceae was introduced. They also showed that *Massarina walkeri* and *A*. *medicaginis* are congeneric and thus introduced a new combination, *A*. *walkeri*. Recently, Jayasiri et al. (2019) introduced another species, *Acrocalymma pterocarpi*, to this family. Dong et al. (2020) introduced the most recent species, *Acrocalymma bipolare*, a freshwater species recovered from submerged wood in the Nile River, Egypt.

Teichosporaceae was established by Barr (2002) based on morphological similarities of *Bertiella*, *Byssothecium*, *Chaetomastia*, *Immotthia*, *Loculohypoxylon*, *Moristroma*, *Sinodidymella*, and the type genus *Teichospora*. However, *Moristroma*, *Byssothecium* and *Bertiella* were excluded from the family by Lumbsch and Huhndorf (2010). Jaklitsch et al. (2016) revised Teichosporaceae and accepted only *Teichospora* in the family. The family Floricolaceae was also synonymized under Teichosporaceae by Jaklitsch et al. (2016), and every genus within this family became a synonym of *Teichospora*. however this current taxonomic rearrangement needs to be verified with wider sampling. In addition subsequent outlines did not follow the monotypic nature of Teichosporaceae (Wijayawardene et al., 2018). Currently, Teichosporaceae contains thirteen accepted genera (Hongsanan et al., 2020a).

The present research paper introduces two new species in Acrocalymmaceae, one new genus and a new species in Monoblastiaceae and one new species in Teichosporaceae from fifteen specimens collected from Honghe, Kunming, Lincang, Qujing, and Yuxi in Yunnan Province, China.

## Materials and Methods

### Isolates and Specimens

Fungal materials were collected from various deciduous trees and dried woody litter in Yunnan Province, China during the dry season. Collected samples were brought to the laboratory in Zip lock plastic bags. Samples were examined with an Olympus SZ61 Series microscope. Single ascospore isolation was carried out following the method described in Senanayake et al. (2020). Germinated spores were individually transferred to potato dextrose agar (PDA) plates and grown at 20°C in daylight. The living cultures were deposited at the Kunming Institute of Botany Culture Collection (KUMCC), Kunming, China, and duplicated at the China General Microbiological Culture Collection Center (CGMCC). Dry herbarium materials have been stored in the herbarium of Cryptogams Kunming Institute of Botany, Academia Sinica (KUN-HKAS). MycoBank numbers have been registered as outlined in MycoBank^1^.

### Morphological Observations

In hand sections of the ascomata/conidiomata, which were mounted in distilled water, the following characteristics were evaluated: ascomata/conidiomata diameter, height, color, and shape; width of peridium; and height and diameter of ostioles. Length and width (at the widest point) of asci, ascospores, conidiophores and conidia were also measured. Images were captured with a Canon EOS 600D digital camera fitted to a Nikon ECLIPSE Ni compound microscope. Measurements were made with the Tarosoft (R) Image Frame Work program, and images used for figures were processed with Adobe Photoshop CS5 Extended version 10.0 software (Adobe Systems, United States).

### DNA Extraction, PCR Amplifications, and Sequencing

Genomic DNA was extracted from the axenic mycelium as described by Phookamsak et al. (2017). When the spores failed to germinate in culture, DNA was extracted directly from the fruiting bodies of the fungus as outlined by Wanasinghe et al. (2018b). DNA to be used as templates for Polymerase chain reaction (PCR) were stored at 4°C for use in regular work and duplicated at -20°C for long-term storage.

The primers and PCR protocols for each gene were conducted by following Thiyagaraja et al. (2020a) and Wanasinghe et al. (2020). PCR was carried out at a volume of 25 μl, which contained 12.5 μl of 2× Power Taq PCR MasterMix (Bioteke Co., China), 1 μl of each primer (10 μM), 1 μl genomic DNA and 9.5 μl deionized water. The amplified PCR fragments were sent to a commercial sequencing provider (BGI, Ltd., Shenzhen, China). The nucleotide sequence data acquired were deposited in GenBank (Table 1).

**TABLE 1:** Taxa used in the phylogenetic analysis of Monoblastiaceae and their corresponding GenBank numbers.

Species	Strain no.	GenBank accession no.				
		ITS	LSU	SSU	*tef*1	mtSSU
*Acrocordia subglobosa*	HTL940	NA	JN887392	JN887373	JN887417	GU327681
*Anisomeridium* cf. *willeyanum*	MPN549	NA	JN887393	NA	NA	JN887407
*Anisomeridium phaeospermum*	MPN539	NA	JN887394	JN887374	NA	NA
*Anisomeridium* sp.	MPN533	NA	JN887395	JN887375	JN887419	NA
*Anisomeridium* sp.	MPN540	NA	JN887397	JN887377	JN887420	JN887409
*Anisomeridium* sp.	MPN534	NA	JN887396	JN887376	NA	JN887408
*Anisomeridium* sp.	MPN542	NA	JN887398	JN887378	NA	JN887410
*Anisomeridium ubianum*	MPN94	NA	GU327709	JN887379	NA	GU327682
*Elsinoe centrolobii*	CBS 222.50	NR_148132	KX886969	NG_062717	DQ677934	NA
*Elsinoe phaseoli*	CBS 165.31	NR_148161	DQ678095	NG_062718	DQ677935	NA
*Eriomyces heveae*	MFLUCC 17-2232	NR_169673	MH109524	NA	NA	NA
*Funbolia dimorpha*	CPC 14170	JF951136	JF951156	JF951136	NA	NA
*Funbolia dimorpha*	CBS 126491	NA	NG_064276	NA	NA	NA
*Heleiosa barbatula*	JK 5548I	NA	GU479787	GU479753	NA	NA
*Italiofungus phillyreae*	CPC 35566	MT223804	MT223899	NA	NA	NA
*Megalotremis verrucosa*	Lucking 26316	NA	GU327718	JN887383	NA	GU327694
*Myriangium hispanicum*	CBS 247.33	KX887304	GU301854	GU296180	GU349055	NA
***Neoheleiosa lincangensis***	**HKAS 111911**	**MW424766**	**MW424781**	**MW424796**	**MW430102**	**MW422272**
***Neoheleiosa lincangensis***	**HKAS 111912**	**MW424765**	**MW424780**	**MW424795**	**MW430101**	**MW422273**
***Neoheleiosa lincangensis***	**HKAS 111913**	**MW424767**	**MW424782**	**MW424797**	**MW430103**	**MW422274**
***Neoheleiosa lincangensis***	**HKAS 111914**	**MW424764**	**MW424779**	**MW424794**	**MW430100**	**MW422275**
*Phellinocrescentia guianensis*	CBS 138913	NR_137935	NG_058119	NA	NA	NA
*Pseudopassalora gouriqua*	CBS 101954	NR_160207	NG_067272	NA	NA	NA
*Pseudopassalora gouriqua*	CPC 1811	NA	JN712565	NA	NA	NA
*Trypetheliopsis kalbii*	MPN243	NA	JN887406	JN887391	NA	GU327703

*The newly generated sequences are indicated in bold. NA: Sequence data not available in GenBank.*

### Molecular Phylogenetic Analyses

#### Sequencing and Sequence Alignment

Sequences generated from different primers were analyzed along with sequences retrieved from GenBank (Tables 1–3). Sequences with high similarity indices were determined from a BLAST search to find the closest matches with taxa in Pleosporales, and from recently published data (Thambugala et al., 2015; Jaklitsch et al., 2016; Jayasiri et al., 2019; Hongsanan et al., 2020b). The multiple alignments of all consensus sequences, as well as the reference sequences were automatically generated with MAFFT v. 7 (Kuraku et al., 2013; Katoh et al., 2019)^2^, and improved manually when necessary using BioEdit v. 7.0.9.0 (Hall, 1999).

**TABLE 2:** Taxa used in the phylogenetic analysis of Acrocalymmaceae and their corresponding GenBank numbers.

Species	Strain no.	GenBank accession no.		
		SSU	LSU	ITS
*Acrocalymma ampeli*	MFLU 19-2734	MW079341	MW063211	MW063150
*Acrocalymma ampeli*	NCYU19-0008	MW079342	MW063212	MW063151
*Acrocalymma aquaticum*	MFLUCC 11-0208	JX276953	NG_042698	NR_121544
*Acrocalymma fici*	CBS 317.76	NA	NG_057056	NR_137953
***Acrocalymma hongheense***	**HKAS 111907**	**MW424792**	**MW424777**	**MW424763**
***Acrocalymma hongheense***	**HKAS 111908**	**MW424791**	**MW424776**	**MW424762**
***Acrocalymma hongheense***	**HKAS 111909**	**MW424790**	**MW424775**	**MW424761**
*Acrocalymma medicaginis*	MFLUCC 17-1439	MT214388	MT214433	MT214339
*Acrocalymma medicaginis*	MFLUCC 17-1423	MT214387	MT214432	MT214338
*Acrocalymma medicaginis*	CPC 24340	NA	KP170713	KP170620
*Acrocalymma pterocarpi*	MFLUCC 17-0926	MK347840	NG_066306	NA
*Acrocalymma pterocarpi*	NC13-171	NA	LC517881	LC517880
*Acrocalymma vagum*	CPC 24226	NA	NA	KP170636
*Acrocalymma vagum*	CPC 24225	NA	NA	KP170635
*Acrocalymma walkeri*	UTHSC DI16-195	NA	LN907338	NA
***Acrocalymma yuxiense***	**HKAS 111910**	**MW424793**	**MW424778**	**NA**
*Ascocylindrica marina*	MD6011	KT252905	KT252907	NA
*Ascocylindrica marina*	MF416	MK007124	MK007123	NA
*Boeremia exigua*	CBS 431.74	EU754084	EU754183	FJ427001

*The newly generated sequences are indicated in bold. NA: Sequence data not available in GenBank.*

**TABLE 3:** Taxa used in the phylogenetic analysis of Teichosporaceae and their corresponding GenBank numbers.

Species	Strain no.	GenBank accession no.				
		ITS	LSU	SSU	*rpb*2	*tef*1
*Asymmetrispora mariae*	C134m	KU601580	KU601580	NA	NA	KU601614
*Asymmetrispora mariae*	C139	KU601582	KU601582	NA	NA	KU601615
*Asymmetrispora mariae*	C144	KU601583	KU601583	NA	NA	KU601613
*Asymmetrispora mariae*	C159	KU601584	KU601584	NA	NA	KU601612
*Asymmetrispora mariae*	CBS 124079	NA	JN851819	NA	NA	KR075166
*Asymmetrispora tennesseensis*	ANM 911	NA	GU385207	NA	NA	GU327769
*Aurantiascoma minimum*	ANM 60	NA	GU385182	NA	NA	NA
*Aurantiascoma minimum*	ANM 933	NA	GU385195	NA	NA	NA
*Aurantiascoma minimum*	GKM 169N	NA	GU385165	NA	NA	GU327768
*Aurantiascoma minimum*	SMH 2448	NA	GU385166	NA	NA	NA
*Floricola clematidis*	MFLUCC 17–2182	MT310638	MT214594	MT226706	NA	MT394651
*Floricola striata*	JK 5603K	NA	GU479785	GU479751	NA	NA
*Floricola striata*	JK 5678I	NA	GU301813	GU296149	GU371758	GU479852
*Floricola viticola*	IT-2178	KT305997	KT305993	KT305995	NA	NA
*Magnibotryascoma acaciae*	CPC 24801	KR611877	KR611898	NA	NA	NA
***Magnibotryascoma kunmingense***	**KUMCC 20-0254**	**MW424769**	**MW424784**	**MW424799**	**MW430112**	**MW430105**
***Magnibotryascoma kunmingense***	**KUMCC 20-0255**	**MW424770**	**MW424785**	**MW424800**	**MW430113**	**MW430106**
***Magnibotryascoma kunmingense***	**KUMCC 20-0256**	**MW424768**	**MW424783**	**MW424798**	**MW430111**	**MW430104**
***Magnibotryascoma kunmingense***	**KUMCC 20-0257**	**MW424771**	**MW424786**	**MW424801**	**MW430114**	**MW430107**
***Magnibotryascoma kunmingense***	**KUMCC 20-0259**	**MW424772**	**MW424787**	**MW424802**	**MW430115**	**MW430108**
***Magnibotryascoma kunmingense***	**KUMCC 20-0260**	**MW424773**	**MW424788**	**MW424803**	**MW430116**	**MW430109**
***Magnibotryascoma kunmingense***	**KUMCC 20-0261**	**MW424774**	**MW424789**	**MW424804**	**MW430117**	**MW430110**
*Magnibotryascoma mali*	MFLUCC 17-0933	MF173433	MF173429	MF173431	MF173437	MF173435
*Magnibotryascoma melanommoides*	MP5	KU601585	KU601585	NA	NA	KU601610
*Magnibotryascoma rubriostiolatum*	C158	KU601587	KU601587	NA	KU601596	KU601607
*Magnibotryascoma rubriostiolatum*	C158x	KU601588	KU601588	NA	KU601597	KU601608
*Magnibotryascoma rubriostiolatum*	TR5	KU601589	KU601589	NA	KU601598	KU601606
*Magnibotryascoma rubriostiolatum*	TR7	KU601590	KU601590	NA	KU601599	KU601609
*Magnibotryascoma* sp.	MFLUCC 12-0088	NA	KF531927	KF531928	NA	NA
*Magnibotryascoma uniseriatum*	ANM 909	NA	GU385206	NA	NA	NA
*Misturatosphaeria aurantonotata*	ATCC 42522	NA	U43479	U43461	AY485625	NA
*Misturatosphaeria aurantonotata*	GKM 1238	NA	GU385173	NA	NA	GU327761
*Misturatosphaeria aurantonotata*	GKM 1280	NA	GU385174	NA	NA	GU327762
*Misturatosphaeria aurantonotata*	SMH 4330	NA	GU385167	NA	NA	GU327770
*Misturatosphaeria* sp.	SMH 3747	NA	GU385196	NA	NA	NA
*Paulkirkia arundinis*	MFLUCC 12-0328	NA	KU848206	NA	NA	NA
*Pseudoaurantiascoma kenyense*	GKM 1195	NA	GU385194	NA	NA	GU327767
*Pseudoaurantiascoma kenyense*	GKM 234N	NA	GU385188	NA	NA	GU327765
*Pseudoaurantiascoma kenyense*	GKM L100Na	NA	GU385189	NA	NA	GU327766
*Ramusculicola clematidis*	MFLUCC 17-2146	MT310640	MT214596	MT226707	MT394707	MT394652
*Ramusculicola thailandica*	MFLUCC 10-0126	KP899138	KP888644	KP899130	NA	KR075170
*Ramusculicola thailandica*	MFLUCC 13-0284	KP899141	KP888647	KP899131	NA	KR075167
*Teichospora auroafricana*	CBS 119330	EU552115	EU552115	NA	NA	NA
*Teichospora auroafricana*	CBS 122674	MH863228	MH874755	NA	NA	NA
*Teichospora claviformis*	GKM 1210	NA	GU385212	NA	NA	GU327763
*Teichospora grandicipis*	CPC 1852	JN712456	JN712520	NA	NA	NA
*Teichospora grandicipis*	CPC 1853	JN712457	JN712521	NA	NA	NA
*Teichospora kingiae*	CPC 29104	KY173468	KY173557	NA	NA	NA
*Teichospora mariae*	C136	KU601581	KU601581	NA	KU601595	KU601611
*Teichospora nephelii*	CPC 27539	KY173469	KY173558	NA	NA	NA
*Teichospora proteae*	CBS 122675	NA	EU552117	NA	NA	NA
*Teichospora pusilla*	C140	KU601586	KU601586	NA	NA	KU601605
*Teichospora quercus*	CBS 143396	MH107920	MH107966	NA	MH108010	MH108030
*Teichospora trabicola*	C134	KU601591	KU601591	NA	KU601600	KU601601
*Teichospora trabicola*	C141	KU601592	KU601592	NA	NA	KU601603
*Teichospora trabicola*	C160	KU601594	KU601594	NA	NA	KU601602
*Torula chromolaenae*	MFLUCC 17-1514	MT214383	MT214477	MT214428	MT235831	MT235792
*Torula chromolaenae*	MFLUCC 17-1504	MT214384	MT214478	MT214429	MT235832	MT235793

*The newly generated sequences are indicated in bold. NA: Sequence data not available in GenBank.*

#### Phylogenetic Analyses

The single-locus datasets were examined for topological incongruences among loci for members of the analyses. The alignments were concatenated into a multi-locus alignment that was subjected to maximum-likelihood (ML) and Bayesian (BI) phylogenetic analyses.

The CIPRES Science Gateway platform (Miller et al., 2010) was used to perform RAxML and Bayesian analyses. ML analyses were made with RAxML-HPC2 on XSEDE v. 8.2.10 (Stamatakis, 2014) using GTR + GAMMA swap model with 1,000 bootstrap repetitions.

Evolutionary models for Bayesian analysis were selected independently for each locus using MrModeltest v. 2.3 (Nylander et al., 2008) under the Akaike Information Criterion (AIC) implemented in both PAUP v. 4.0b10 and GTR + I + G was selected as the best fit model for all three analyses. MrBayes analyses were performed setting GTR + I + G, 5 M generations, sampling every 1,000 generations, ending the run automatically when standard deviation of split frequencies dropped below 0.01 with a burn-in fraction of 0.25. ML bootstrap values equal or greater than 60% and BYPP greater than 0.95 are given above each node of every trees.

Phylograms were visualized with FigTree v1.4.0 program (Rambaut, 2014) and reorganized in Microsoft power point (2007) and Adobe Illustrator^®^ CS5 (Version 15.0.0, Adobe^®^, San Jose, CA). The finalized alignments and trees were deposited in TreeBASE, submission ID: 27599^3^.

## Results

### Phylogenetic Analyses

**FIGURE 1:**
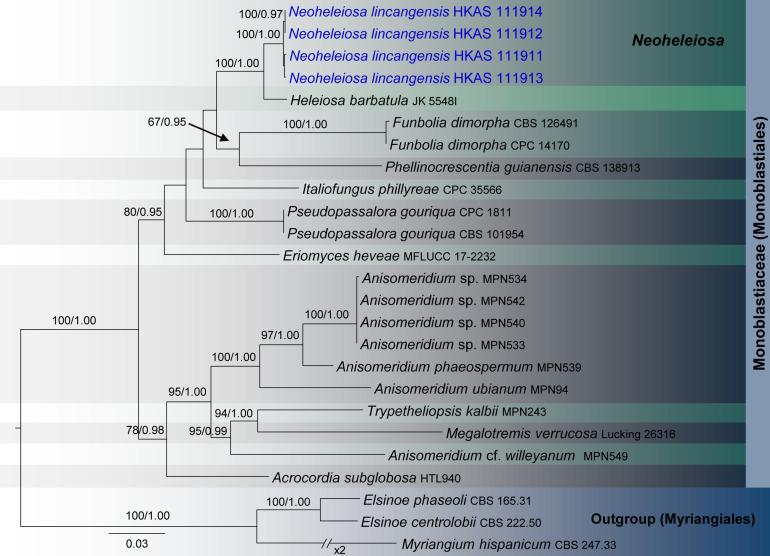
RAxML tree based on a combined dataset of partial SSU, LSU, ITS, *tef*1, and mtSSU sequence analyses in Monoblastiaceae. Bootstrap support values for ML equal to or greater than 60%, Bayesian posterior probabilities (BYPP) equal to or greater than 0.95 are shown as ML/BI above the nodes. The new isolates are in blue. The scale bar represents the expected number of nucleotide substitutions per site.

**FIGURE 2:**
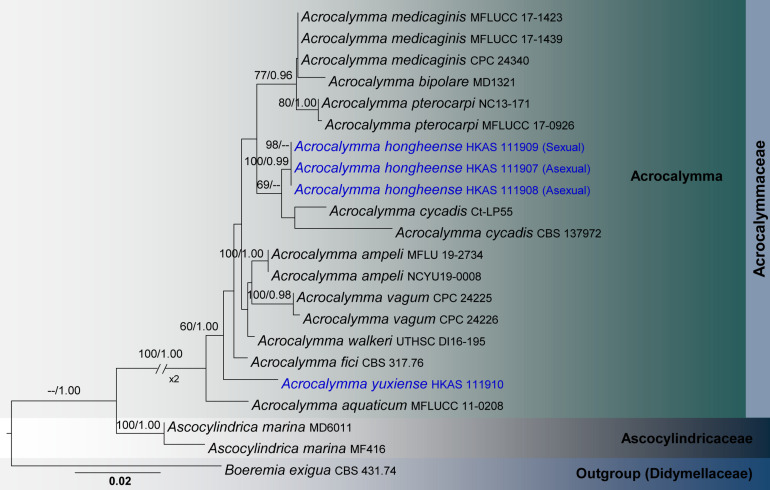
RAxML tree based on a combined dataset of partial SSU, LSU and ITS sequence analyses in Acrocalymmaceae. Bootstrap support values for ML equal to or greater than 60%, Bayesian posterior probabilities (BYPP) equal to or greater than 0.95 are shown as ML/BI above the nodes. The new isolates are in blue. The scale bar represents the expected number of nucleotide substitutions per site.

**FIGURE 3:**
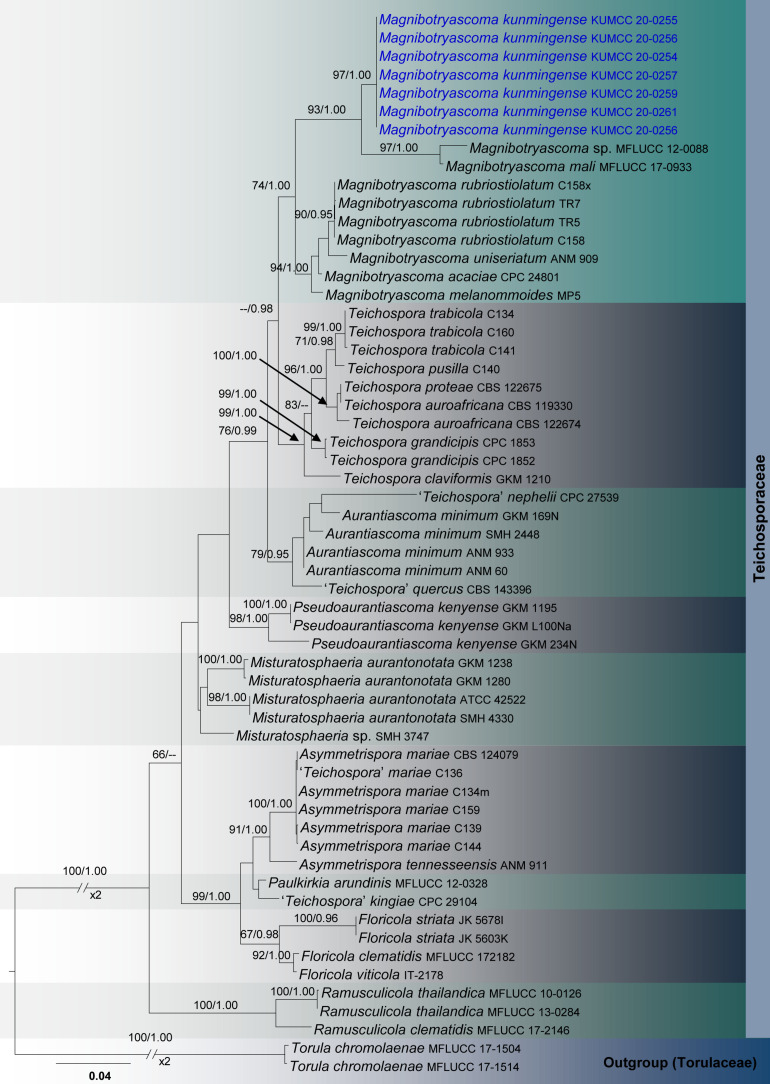
RAxML tree based on a combined dataset of partial SSU, LSU, ITS, *tef*1, and *rpb*2 sequence analyses in Teichosporaceae. Bootstrap support values for ML equal to or greater than 60%, Bayesian posterior probabilities (BYPP) equal to or greater than 0.95 are shown as ML/BI above the nodes. The new isolates are in blue. The scale bar represents the expected number of nucleotide substitutions per site.

Three analyses were performed in this study; the first is an updated phylogeny of the genera in Monoblastiaceae (Figure 1), whereas the remaining two datasets represent taxa in Acrocalymmaceae (Figure 2) and genera treated in Teichosporaceae (Figure 3), respectively.

Monoblastiaceae SSU, LSU, ITS, *tef* 1, and mtSSU phylogeny (Figure 1): The alignment contained 25 isolates and the tree was rooted to *Elsinoe centrolobii* (CBS 222.50) and *E*. *phaseoli* (CBS 165.31). The final alignment contained a total of 4,062 characters used for the phylogenetic analyses, including alignment gaps. The RAxML analysis of the combined dataset yielded a best scoring tree with a final ML optimization likelihood value of −14276.596302. The matrix had 1,076 distinct alignment patterns, with 49.73% of undetermined characters or gaps. Parameters for the GTR + I + G model of the combined amplicons were as follows: Estimated base frequencies; *A* = 0.249541, *C* = 0.238483, *G* = 0.280978, *T* = 0.230998; substitution rates *AC* = 0.975103, *AG* = 2.399794, *AT* = 1.850660, *CG* = 1.423793, *CT* = 6.985604, *GT* = 1.000; proportion of invariable sites *I* = 0.411774; gamma distribution shape parameter α = 0.563137. The Bayesian analysis ran 125,000 generations before the average standard deviation for split frequencies reached below 0.01 (0.008726). The analysis generated 1,251 trees (saved every 100 generations) from which 939 were sampled after 25% of the trees were discarded as burn-in. The alignment contained a total of 1,077 unique site patterns.

Acrocalymmaceae SSU, LSU, and ITS phylogeny (Figure 2): The alignment contained 22 isolates and the tree was rooted to *Boeremia exigua* (CBS 431.74). The final alignment contained a total of 2,317 characters used for the phylogenetic analyses, including alignment gaps. The RAxML analysis of the combined dataset yielded a best scoring tree with a final ML optimization likelihood value of −5083.5038. The matrix had 265 distinct alignment patterns, with 31.12% of undetermined characters or gaps. Parameters for the GTR + I + G model of the combined amplicons were as follows: Estimated base frequencies; *A* = 0.250179, *C* = 0.212903, *G* = 0.27372, *T* = 0.263198; substitution rates *AC* = 2.467522, *AG* = 3.254238, *AT* = 3.774672, *CG* = 0.816185, *CT* = 11.170637, *GT* = 1.000; proportion of invariable sites *I* = 0.737361; gamma distribution shape parameter α = 0.95506. The Bayesian analysis ran 420,000 generations before the average standard deviation for split frequencies reached below 0.01 (0.009821). The analysis generated 4,201 trees (saved every 100 generations) from which 3,151 were sampled after 25% of the trees were discarded as burn-in. The alignment contained a total of 266 unique site patterns.

Teichosporaceae SSU, LSU, ITS, *tef*1, and *rpb*2 phylogeny (Figure 3): The alignment contained 58 isolates and the tree was rooted to *Torula chromolaenae* (MFLUCC 17-1504 and MFLUCC 17-1514). The final alignment contained a total of 4,474 characters used for the phylogenetic analyses, including alignment gaps. The RAxML analysis of the combined dataset yielded a best scoring tree with a final ML optimization likelihood value of −16749.008095. The matrix had 1,159 distinct alignment patterns, with 47.5% of undetermined characters or gaps. Parameters for the GTR + I + G model of the combined amplicons were as follows: Estimated base frequencies; *A* = 0.242588, *C* = 0.25624, *G* = 0.278572, *T* = 0.222599; substitution rates *AC* = 1.47144, *AG* = 3.993923, *AT* = 1.82804, *CG* = 1.343882, *CT* = 11.37556, *GT* = 1.000; proportion of invariable sites *I* = 0.495477; gamma distribution shape parameter α = 0.4815. The Bayesian analysis ran 390,000 generations before the average standard deviation for split frequencies reached below 0.01 (0.009894). The analysis generated 3,901 trees (saved every 100 generations) from which 2,926 were sampled after 25% of the trees were discarded as burn-in. The alignment contained a total of 1,162 unique site patterns.

The phylogenetic results obtained for each dataset are discussed where applicable in the descriptive notes below.

## Taxonomy

In the present study, one new genus and four new species were found. These taxa are subsequently described in the orders Monoblastiales and Pleosporales below.

**Monoblastiaceae** Walt. Watson, New Phytologist 28: 106 (1929)

### Notes

Hongsanan et al. (2020b) accepted 11 genera viz. *Acrocordia*, *Anisomeridium*, *Caprettia*, *Eriomyces*, *Funbolia*, *Heleiosa*, *Megalotremis*, *Monoblastia*, *Phellinocrescentia*, *Pseudopassalora*, and *Trypetheliopsis* in Monoblastiaceae. Meanwhile, Crous et al. (2020) added *Italiofungus* to accommodate *Italiofungus phillyreae*, which was collected on leaves of *Phillyrea latifolia* from Italy. In this study, we add another genus, *Neoheleiosa* gen. nov. to Monoblastiaceae based on multi-gene phylogenetic evidences.

***Neoheleiosa* Mortimer gen. nov.**
MycoBank: MB838517
Etymology: The generic epithet reflects the similarity to *Heleiosa*.

*Saprobic* on dead wood. **Sexual morph:** *Ascomata*, solitary, scattered, immersed to erumpent, globose to subglobose or obpyriform, dark brown to black, coriaceous, ostiolate. *Ostiole* centric or eccentric, papillate, black, smooth, filled with hyaline cells when mature. *Peridium* comprising blackish to dark brown, thin-walled cells of *textura globulosa*. *Hamathecium* comprising numerous, branched, septate, pseudoparaphyses. *Asci* 8-spored, bitunicate, fissitunicate, cylindric, pedicellate, rounded, and thick-walled at the apex, with an ocular chamber. *Ascospores* overlapping uniseriate, narrowly ovoid to clavate, 1-septate, initially hyaline, becoming dark brown at maturity, with conically rounded ends, guttulate, thick walled, faintly longitudinally striated, lacking a mucilaginous sheath. **Asexual morph:** Undetermined.

**FIGURE 4:**
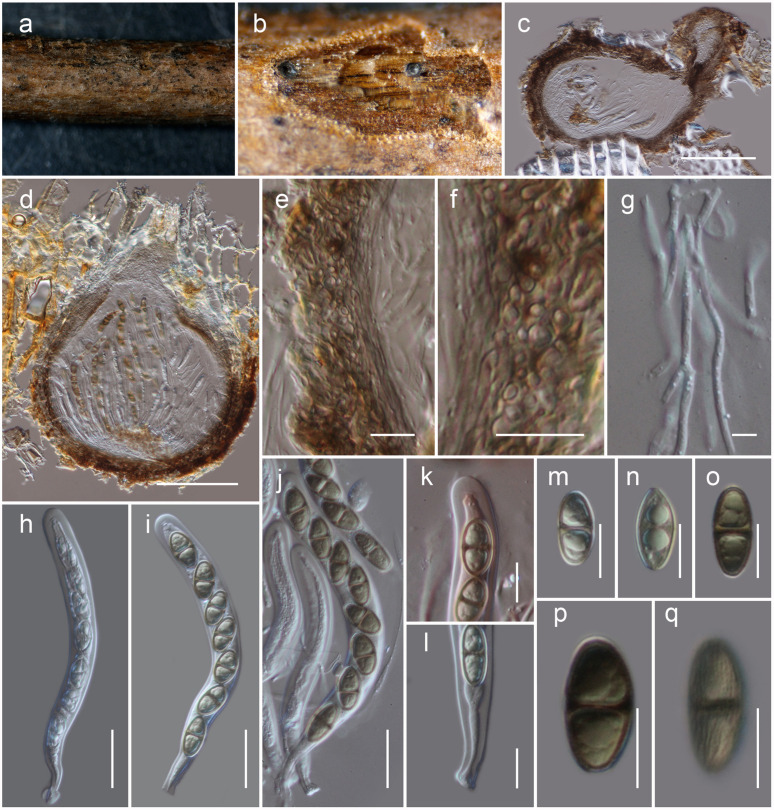
*Neoheleiosa lincangensis* (HKAS 111914). **(a)** Ascomata observed on host substrate. **(b,d)** Horizontal sections of ascomata. **(c)** Vertical sections through an ascoma. **(e,f)** Cells of peridium. **(g)** Pseudoparaphyses. **(h–j)** Asci. **(k)** Ocular chamber. **(l)** Pedicel. **(m–q)** Ascospores (note: **q** longitudinally striated). Scale bars: **(c,d)** = 100 μm, **(e, f, k–q)** = 10 μm, **(g)** = 5 μm, **(h–j)** = 20 μm.

Type species: *Neoheleiosa lincangensis*
***Neoheleiosa lincangensis* Mortimer sp. nov.** Figure 4.
MycoBank: MB838518
Etymology: The specific epithet reflects Lincang, from where the holotype was collected.
Holotype: HKAS 111914

*Habitat* terrestrial, s*aprobic* on dead twigs of *Pittosporum* sp. **Sexual morph:** *Ascomata*, 130–160 μm high, 200–250 μm diam. ($\bar{x}$ = 146 × 217 μm, *n* = 5), solitary, scattered, immersed to erumpent, globose to subglobose or obpyriform, dark brown to black, coriaceous, ostiolate. *Ostiole* 70–110 μm long, 30–50 μm diam. ($\bar{x}$ = 90 × 40 μm, *n* = 5), eccentric, papillate, black, smooth, filled with hyaline cells when mature. *Peridium* 8–12 μm thick at the base, 15–25 μm wide at sides, comprising blackish to dark brown, thin-walled cells of *textura globulosa*. *Hamathecium* 2–2.5 μm wide, comprising numerous, filamentous, branched, septate, pseudoparaphyses. *Asci* 115–135 × 10–12 μm ($\bar{x}$ = 124 × 10.6 μm, *n* = 30), 8-spored, bitunicate, fissitunicate, cylindrical, pedicel furcate, rounded and thick-walled at the apex, with an ocular chamber. *Ascospores* 16.5–17.5 × 7–9 μm ($\bar{x}$ = 17.1 × 8.2 μm, *n* = 30), overlapping uniseriate, narrowly ovoid to clavate, 1-septate, initially hyaline, becoming dark brown at maturity, with conically rounded ends, guttulate, thick walled, faintly longitudinally striated, lacking a mucilaginous sheath. **Asexual morph:** Undetermined.

#### Material Examined

**China**, Yunnan Province, Lincang, Yongde County, Bankaxiang, (N: 23.997479, E: 99.480670), on dead twigs of *Pittosporum* sp., 8 April 2019, D.N. Wanasinghe, DW0738-07 (HKAS 111914, holotype); *ibid*. DW0738-09 (HKAS 111911); DW0738-11 (HKAS 111912); DW0738-12 (HKAS 111913).

### Notes

Four specimens with 1-septate, brown ascospores species were collected on dead twigs of *Pittosporum* from Bankaxiang in Yunnan Province. We made several attempts to obtain a culture via single spore isolations and direct isolation from fungal tissues (Senanayake et al., 2020). However, we were unable to get a pure culture, and DNA was extracted directly from the fruiting bodies. Sequence data of these four collections grouped in a strongly supported monophyletic clade in the concatenated SSU, LSU, ITS, *tef* 1 and mtSSU sequence analyses (Figure 1). Morphologically all these collections were identical and there were no differences in their DNA sequence data. Thus, we recognize that all of these specimens belong to a single species, *Neoheleiosa lincangensis* sp. nov.

**FIGURE 5:**
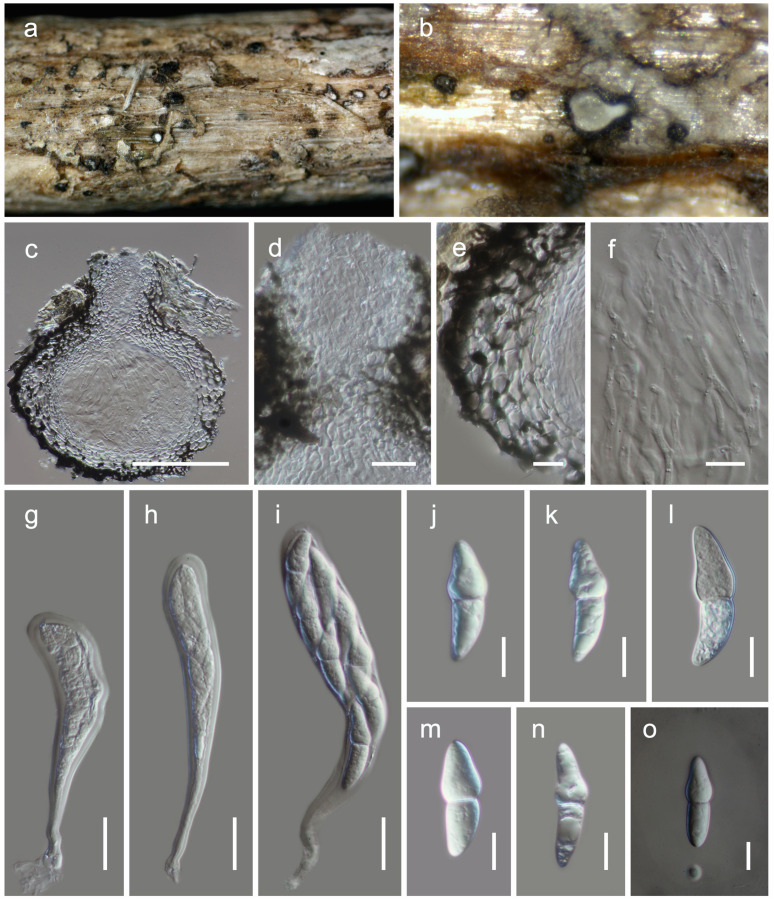
Sexual morph of *Acrocalymma hongheense* (HKAS 111909). **(a)** Ascomata observed on host substrate. **(b)** Horizontal section of an ascoma. **(c)** Vertical sections through an ascoma. **(d)** Close up of ostiole. **(e)** Cells of peridium. **(f)** Pseudoparaphyses. **(g–i)** Asci. **(i–o)** Ascospores (note: **o** stained with Indian Ink). Scale bars: **(c)** = 50 μm, **(d,g–i)** = 20 μm, **(e,f,j–o)** = 10 μm.

**FIGURE 6:**
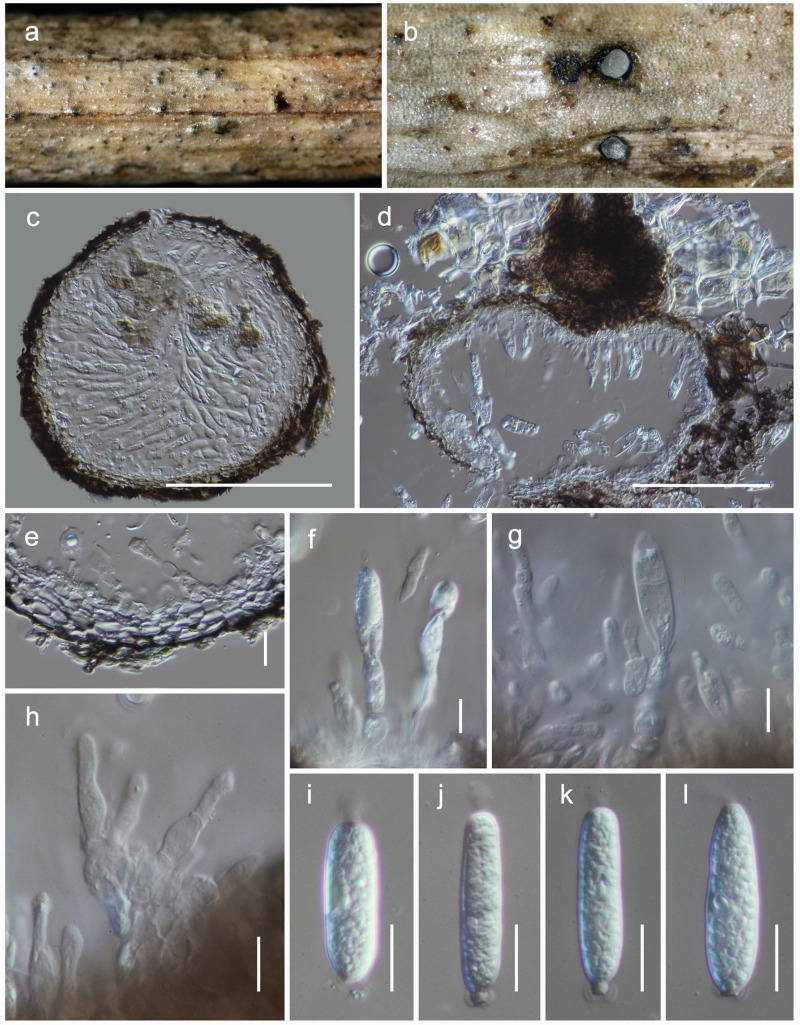
Asexual morph of *Acrocalymma hongheense* (HKAS 111908). **(a)** Conidiomata observed on host substrate. **(b)** Horizontal section of a conidioma. **(c,d)** Vertical sections through conidiomata. **(e)** Cells of pycnidia wall. **(f–h)** Conidiogenous cells. **(i–l)** Conidia (note the mucoid mass oozing out from apex of all conidia). Scale bars: **(c,d)** = 100 μm, **(e–l)** = 10 μm.

**Acrocalymmaceae** Crous and Trakun., IMA Fungus 5 (2): 404 (2014)
***Acrocalymma hongheense* Mortimer sp. nov.** Figures 5, 6.
MycoBank: MB838519
Etymology: The specific epithet reflects Honghe, from where the holotype was collected.
Holotype: HKAS 111909

*Habitat* terrestrial, saprobic on dead woody litter. **Sexual morph:** *Ascomata* 180–220 μm high, 160–200 μm diam. ($\bar{x}$ = 196 × 184 μm, *n* = 5), dark brown, gregarious, immersed beneath host epidermis, visible as numerous, raised, dome-shaped areas on host surface, globose to subglobose, uni-loculate, glabrous with rough walls, coriaceous, ostiolate. *Ostioles* 50–80 μm long, 30–50 μm diam. ($\bar{x}$ = 70 × 40 μm, *n* = 5), centrally located, filled with hyaline cells. *Peridium* 25–40 μm wide, of unequal thickness, thick on sides toward the apex, composed of dark brown to black cells, arranged in *textura angularis*. *Hamathecium* composed of 1–2.5 μm wide, numerous, filamentous, branched, septate, pseudoparaphyses. *Asci* 100–140 × 15–22 μm ($\bar{x}$ = 118.9 × 18.5 μm, *n* = 30), 8-spored, bitunicate, cylindric-clavate, with a furcate to truncate pedicel, apically rounded with an ocular chamber. *Ascospores* 25–35 × 9.5–11 μm ($\bar{x}$ = 31.8 × 9.8 μm, *n* = 40), overlapping bi-seriate, hyaline, fusiform with acute ends, 1-septate, constricted at the septum, upper cell wider than lower cell, smooth-walled, surrounded by a thick, distinct sheath. **Asexual morph:** coelomycetous. *Conidiomata* 150–200 μm high, 180–220 μm diam. ($\bar{x}$ = 166 × 214 μm, *n* = 5), pycnidial, dark brown, immersed to semi-erumpent, globose, with a central ostiole. *Pycnidia wall* 10–20 μm wide, of unequal thickness, composed of dark brown to black cells, arranged in *textura angularis*. *Conidiophores* reduced to conidiogenous cells or with a single supporting cell. *Conidiogenous cells* 8–17 × 3.5–7 μm ($\bar{x}$ = 11.5 × 5.3 μm, *n* = 40), lining the inner cavity, subcylindrical, hyaline, smooth, annelledic, proliferating percurrently at apex, with prominent periclinal thickening at the apex. *Conidia* 20–35 × 7–9 μm ($\bar{x}$ = 27.3 × 7.5 μm, *n* = 40), hyaline, smooth, guttulate, solitary, subcylindrical, straight, obtusely rounded and with mucoid ooze at the apex, protuberant and with a rounded hilum at base, aseptate, guttulate, sometimes conidia becoming 1-septate.

#### Material Examined

**China**, Yunnan Province, Honghe Hani and Yi Autonomous Prefecture, Mengzi, (N: 23.185677, E: 103.413816), on woody litter, 16 March 2019, D.N. Wanasinghe, DW0375-004 (HKAS 111909, holotype); *ibid*. DW0375-005 (HKAS 111908); *ibid*. Yuxi, Yi and Dai Autonomous County, Yuanjiang Hani, (N: 23.740730, E: 103.413816), 24 May 2019, DW0636-014 (HKAS 111907).

### Notes

During our investigation on the diversity of woody-based Dothideomycetes in Yunnan Province, three isolates (HKAS 111907, HKAS 111908, HKAS 111909) were recovered from decaying woody litter in Honghe and Yuxi counties. Two of them had asexual morphs while the third specimen had a sexual morph. Conidiomata, conidiophores and conidia of these asexual fungi morphologically resemble the remaining taxa in *Acrocalymma* (Alcorn and Irwin, 1987; Zhang et al., 2012; Crous et al., 2014; Trakunyingcharoen et al., 2014). The sexual morph was similar to *Acrocalymma pterocarpi*, which was the only known sexual morph in the genus, by its ascomata and asci features (Jayasiri et al., 2019). Phylogenetically, HKAS 111907, HKAS 111908, and HKAS 111909 are monophyletic with strong bootstrap supports (100% ML/0.99 BYPP, Figure 2). This clade has a sister relationship to *Acrocalymma cycadis* (CBS 137972 and Ct-LP55), but was not statistically supported. Within our new isolates, there were no nucleotide differences in ITS, LSU and SSU gene regions. Therefore, we recognize these three isolates belong to one species (Jeewon and Hyde, 2016), which we introduce as a new species herein.

**FIGURE 7:**
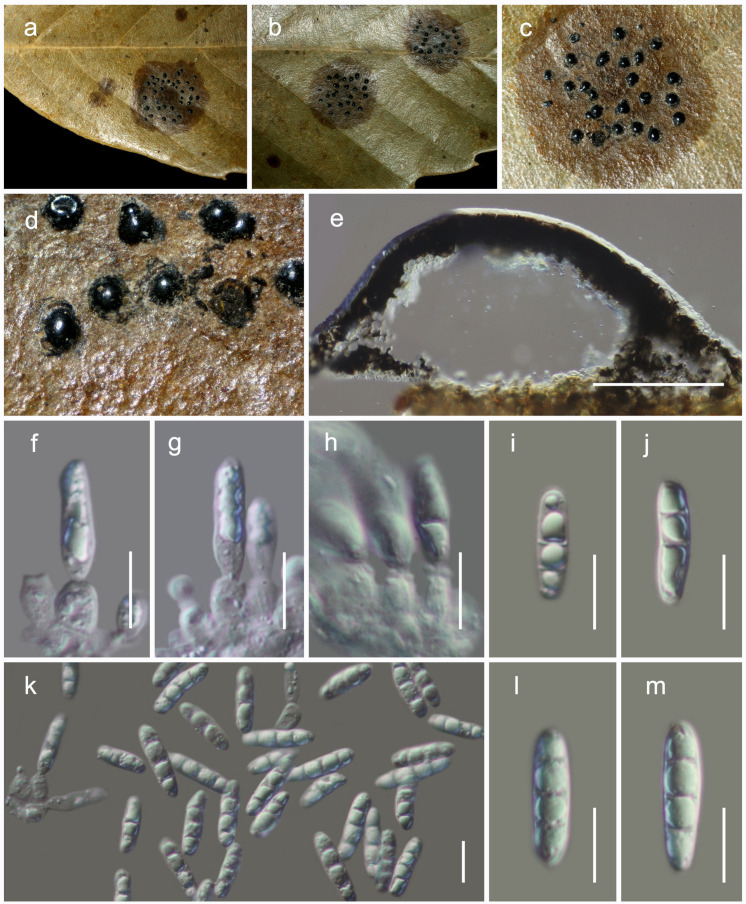
*Acrocalymma yuxiense* (HKAS 111910). **(a)** Conidiomata observed on dried leaves of *Quercus* sp. **(e)** Vertical sections through a conidioma. **(f–h)** Conidiogenous cells. **(i–m)** Conidia. Scale bars: **(e)** = 100 μm, **(f–m)** = 10 μm.

***Acrocalymma yuxiense* Mortimer sp. nov.** Figure 7.
MycoBank: MB838520
Etymology: The specific epithet reflects Yuxi, from where the holotype was collected.
Holotype: HKAS 111910

*Habitat* terrestrial, saprobic or weakly pathogenic on dried leaves of *Quercus* sp. **Sexual morph:** Undetermined. **Asexual morph:** coelomycetous, *Conidiomata* 130–170 μm high, 220–260 μm diam. ($\bar{x}$ = 155 × 242 μm, *n* = 5), pycnidial, dark brown, immersed to semi-erumpent, globose, with central ostiole. *Pycnidia wall* 10–30 μm wide, of unequal thickness, composed of dark brown to black cells, arranged in *textura angularis*. *Conidiophores* reduced to conidiogenous cells. *Conidiogenous cells* 4–8 × 2.5–4.5 μm ($\bar{x}$ = 6.3 × 3.4 μm, *n* = 20), lining the inner cavity, cylindrical to subcylindrical, hyaline, smooth, percurrently proliferating 1–2 times at apex, with prominent periclinal thickening at apex. *Conidia* 15–21 × 4–5 μm ($\bar{x}$ = 18.4 × 4.6 μm, *n* = 30), hyaline, smooth, guttulate, solitary, subcylindrical, straight, obtusely rounded at apex and base, 3-euseptate, guttulate, conidia sometimes 1-septate.

#### Material Examined

**China**, Yunnan Province, Yuxi (N: 24.210342, E: 102.540022), on dried leaves of *Quercus glauca*, 14 March 2019, D.N. Wanasinghe, DW0886-01 (HKAS 111910, holotype).

### Notes

*Acrocalymma yuxiense*, collected from dried leaves of *Quercus glauca* in Yunnan, is in an independent lineage with weak support and is phylogenetically distinct from other extant species of *Acrocalymma* (Figure 2). This new species differs from other taxa in *Acrocalymma* in having conidia with 3 vertical eusepta while other species produce aseptate conidia.

**FIGURE 8:**
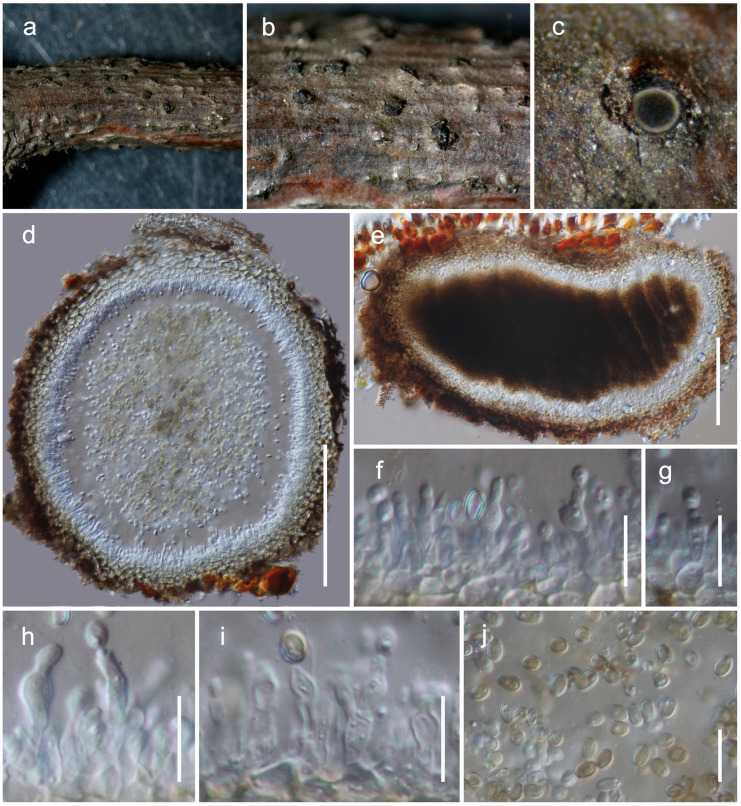
*Magnibotryascoma kunmingense* (HKAS 111919). **(a,b)** Conidiomata observed on host **(c)** Horizontal section of a conidioma. **(e)** Vertical sections through a conidioma. **(f–i)** Conidiogenous cells. **(j)** Conidia. Scale bars: **(d,e)** = 100 μm, **(f–j)** = 10 μm.

**Teichosporaceae** M.E. Barr, Mycotaxon 82: 374 (2002)
***Magnibotryascoma kunmingense* Mortimer sp. nov.** Figure 8
MycoBank: MB838522
Etymology: The specific epithet reflects Kunming, from where the holotype was collected.
Holotype: HKAS 111919

*Habitat* terrestrial, saprobic on dead twigs. **Sexual morph:** Undetermined. **Asexual morph:** coelomycetous, *Conidiomata* 150–180 μm high, 250–380 μm diam. ($\bar{x}$ = 161 × 323 μm, *n* = 5) pycnidial, solitary, aggregated, uniloculate, immersed, globose to subglobose, coriaceous, dark brown to brown, papillate, with a central ostiole. *Pycnidia wall* 20–30 μm wide, thick, 2-layered, with outer layer composed of light brown to brown cells of *textura angularis*, lined with a hyaline innermost layer bearing conidiogenous cells. *Conidiophores* reduced to conidiogenous cells. *Conidiogenous cells* 3–7 × 2–4.5 μm ($\bar{x}$ = 4.9 × 3.1 μm, *n* = 20), enteroblastic, annelledic, discrete, cylindrical to oblong, hyaline, arising from the inner layer of pycnidium wall. *Conidia* 3.8–5.1 × 2.5–3.4 μm ($\bar{x}$ = 4.4 × 3 μm, *n* = 30), subglobose, oval, guttulate, hyaline when immature, pale brown at maturity, aseptate, smooth-walled.

#### Culture Characteristics

*Colonies grew on PDA* at 20°C in the dark reached 2 cm diam., within 14 days, dense, circular, slightly raised, surface smooth, entire margin, white in surface view and off-white to gray in reverse.

#### Material Examined

**China**, Yunnan Province, Kunming, Panlong District, (N: 25.139854, E: 102.737896), on dead twigs of *Machilus yunnanensis* Lecomte, 26 June 2020, D.N. Wanasinghe, DWKIB20-013B (HKAS 111919, holotype), ex-type culture (KUMCC 20-0254); *ibid*. *Acer cappadocicum* var. *sinicum* Rehd., DWKIB20-039 (HKAS 111916), culture (KUMCC 20-0255); *ibid*. DWKIB20-027 (HKAS 111921), culture (KUMCC 20-0256); *ibid*. DWKIB20-041 (HKAS 111915), culture (KUMCC 20-0257); *ibid*. Qujing, Luoping County, Changdixiang (N: 25.018503, E: 104.406763), DW1303-1 (HKAS 111917), culture (KUMCC 20-0261); *ibid*. Kunming, Xishan, (N: 25.043763, E: 102.482118), 18 July 2019, DW1287-9 (HKAS 111920), culture (KUMCC 20-0259); *ibid*. Kunming, Xishan, (N: 25.119848, E: 102.546979), 19 July 2019, DW1344-5 (HKAS 111918), culture (KUMCC 20-0260).

### Notes

Seven isolates of *Magnibotryascoma kunmingense* were obtained from *Acer cappadocicum* var. *sinicum*, *Machilus yunnanensis* and decaying woody litter in Kunming (Panlong and Xishan) and Qujing (Changdixiang). All these specimens were morphologically similar to *Magnibotryascoma mali* in terms of their conidial and conidiomatal characteristics. Phylogenetically, all strains of *Magnibotryascoma kunmingense* are monophyletic with 97% ML and 1.00 BI statistical support values (Figure 3). This clade constitutes a sister clade to *Magnibotryascoma* sp. (MFLUCC 12-0088) and *M*. *mali* (MFLUCC 17-0933).

## Discussion

The mountainous region of Yunnan Province, China is an important global biodiversity hotspot for studying the evolution of plants, animals, and fungi (Feng and Yang, 2018). The mountains of Southwest China; Eastern Himalaya-Nepal-India and Indo-Burma-India-Myanmar are included in the world’s 35 biodiversity hotspots, and these three hotspot regions intersect in Yunnan Province (Myers et al., 2000; Mittermeier et al., 2005). As a result of the diverse landscape and climatic conditions within Yunnan Province, fungi in Yunnan Province have higher rates of endemism; however, they also share evolutionary connections with species from other regions of the world (Feng and Yang, 2018). Among them, wood-decaying Basidiomycota in tropical China are well studied, and many new species have been documented (Dai et al., 2003, 2004, 2011; Cui et al., 2009; Wang et al., 2011; Yuan and Dai, 2012). This has facilitated better understanding of species diversity and the systematics of woody-based basidomycetous groups, such as Polyporales. However, woody-based microfungi such as Dothideomycetes are relatively neglected compared to the level of research conducted on Basidiomycetes (Wanasinghe et al., 2020). In the last few years, there has been increasing attention on woody-based microfungal occurrences, and more studies are reporting on the microfungal diversity, especially in Dothideomycetes, of Yunnan Province (Bao et al., 2018; Huang et al., 2018; Luo et al., 2018; Wanasinghe et al., 2018a, 2020, 2021; Rathnayaka et al., 2019; Qiao et al., 2020; Thiyagaraja et al., 2020b; Yasanthika et al., 2020).

In this study, we added taxonomic novelties in Acrocalymmaceae, Monoblastiaceae, and Teichosporaceae from Yunnan Province. To the best of our knowledge, these are the first accounts of the species in these three families from Yunnan Province. Within the broader region of China, there is one report of *Acrocalymma medicaginis* (Acrocalymmaceae) on *Trachycarpus fortunei* (Taylor and Hyde, 2003) and seven species from Teichosporaceae viz. *Magnibotryascoma mali* (on *Malus halliana*), *Sinodidymella verrucose* (on *Salsola gemmascens*), *Teichospora borealis* (on *Salix tianschanica*), *T*. *lonicerae* (on *Lonicera stenanthera*), *T*. *populi* (on *Populus* sp.), *T*. *solitaria* (on *Nitraria sibirica*), and *T*. *winteriana* (on *Hippophae rhamnoides*) currently documented (Yuan and Barr, 1995; Teng, 1996; Zhuang, 2005; Zhang et al., 2012; Hyde et al., 2017).

*Neoheleiosa* has a close phylogenetic affinity to *Heleiosa* with 100% ML and 1.00 BI support values (Figure 1). Kohlmeyer et al. (1996) introduced *Heleiosa* as a monotypic genus to accommodate *Heleiosa barbatula*, which is characterized by cylindrical asci with a short pedicel, 1-septate, ellipsoid ascospores with appendages. This fungus was found on senescent leaves of *Juncus* in salt marshes on the Atlantic coast of the United States (North Carolina, Virginia). Ascomata, asci and ascospore shapes of both genera were shown to be similar. However, the ascospores of *Neoheleiosa* do not have any appendages (Figures 4m–q), whereas *Heleiosa* have 10 or more short and curved hair-like subapical appendages at each end. Ascospores with a gelatinous sheath or appendages may help fungi to attach to plant substrates in aquatic or marine habitats (Shearer, 1993; Hyde and Goh, 2003; Jones, 2006; Devadatha et al., 2018; Hashimoto et al., 2018). The subapical appendages of *Heleiosa* ascospores could be an adaptation for its marine-based habitat and loss of appendaged ascospores in *Neoheleiosa* is potentially advantageous to adaptation to a non-aquatic habitat.

The surface ornamentation of spores, such as the presence of or absence of appendages, is often used in ascomycetous taxonomy to delineate species or genera (Jeewon et al., 2003; Liu et al., 2014; Phookamsak et al., 2014; Wijayawardene et al., 2016; Paz et al., 2017; Réblová et al., 2015, 2018; Pem et al., 2019b). Abbott and Currah (1997) and Villegas et al. (2005) reported spore ornamentation as a useful character in differentiating various genera within Helvellaceae and Gomphales. Jeewon et al. (2002) clearly demonstrated that species bearing appendages can form distinct phylogenetic lineages and discussed how this character can be a significant phylogenetic marker at the intergeneric level. Considering the similarities shown between our newly proposed genus and *Heleiosa*, we based our placement of these specimens in a new genus due to the lack of any appendages on the ascospores, a significant variation in morphology compared to the spores of *Heleiosa*. The ascospores of *Neoheleiosa* are ornamented with longitudinal streaks (Figure 4q), whereas *Heleiosa* have smooth-walled ascospores. Furthermore, the arrangement of peridium cells has been reported to be useful to demarcate different genera (Hyde et al., 2013; Tian et al., 2015; Ekanayaka et al., 2017; Paz et al., 2017; Senanayake et al., 2018). In our study, we observed that the peridium cells of *Heleiosa* are *textura angularis*, while *Neoheleiosa* have cells of *textura globulosa*. It could be possible that with wider collections in the future, species from these two genera will constitute distinct lineages that will further substantiate their generic placement, a scenario which has been reported in other studies (Huang et al., 2017; Wanasinghe et al., 2018b; Jaklitsch and Voglmayr, 2020).

Acrocalymmaceae includes a single genus with eight species viz. *Acrocalymma aquaticum*, *A*. *bipolare*, *A*. *cycadis*, *A*. *fici*, *A*. *medicaginis*, *A*. *pterocarpi*, *A*. *vagum* and *A*. *walker*. These are reported from terrestrial habitats, excluding *Acrocalymma aquatica* and *A*. *bipolare* (Zhang et al., 2012; Dong et al., 2020). The majority of these species are saprobic on various host substrates (Hongsanan et al., 2020a,b; Tennakoon et al., 2021). Only *Acrocalymma medicaginis* and *A. vagum* are reported as pathogens (Crous et al., 2014; Trakunyingcharoen et al., 2014). Moreover, *Acrocalymma medicaginis* is known as the causal agent of root and crown rot of *Medicago sativa* (Alcorn and Irwin, 1987). Even though there are only eight described species, more than 200 ITS sequences have been deposited from these species in GenBank. Endophytic strains of *Acrocalymma vagum* have the highest number of reported sequences, followed by *A*. *medicaginis*.

Thambugala et al. (2015) introduced *Magnabotrioscoma* to accommodate *M*. *uniseriata* (≡*Misturatosphaeria uniseriate*), which is morphologically distinct from the remaining genera in Floricolaceae. *Magnabotrioscoma* species have been reported as woody-based saprobes on *Clematis vitalba, Malus halliana, Ribes sanguineum*, *Robinia pseudoacacia*, *Salix* sp., and *Vaccinium myrtillus* from Belgium, China, Germany, Norway and the United Kingdom (Jaklitsch et al., 2016; Hyde et al., 2017; Phukhamsakda et al., 2020). In this study, we classified another species in this genus, *Magnibotryascoma kunmingensis*, growing on the decaying woody litter of *Acer cappadocicum* var. *sinicum* and *Machilus yunnanensis*. The sexual morph of the genus is characterized by erumpent to superficial ascomata lacking a subiculum and fusiform to elliptical and guttulate ascospores (Mugambi and Huhndorf, 2009; Thambugala et al., 2015; Phukhamsakda et al., 2020), and the asexual morph has pycnidial conidiomata featuring aseptate and brown conidia (Crous et al., 2015; Jaklitsch et al., 2016). One interesting finding in this genus is the close connection between uncultured fungal strains (i.e., JF945459, KC978028, KX515906, LS994072) and endophytic strains (i.e., EF419935, EF419972, EF419996) with our new strains in BLAST search results. These strains are not linked to morphological details, and it is therefore difficult to provide further insights into their morpho-phylo relationships. This study reveals that there are potentially many new species awaiting discovery in this region (Hyde et al., 2018).

## Data Availability Statement

The datasets generated for this study can be found in the NCBI GenBank, MycoBank and TreeBASE.

## Author Contributions

PM, DW, and RJ designed the study. DW did the sample collection. PM and DW were involved in the phylogenetic analyses. SL and J-CX contributed for the research funds. All authors contributed to the writing, preparation, and submission of the manuscript.

## Conflict of Interest

The authors declare that the research was conducted in the absence of any commercial or financial relationships that could be construed as a potential conflict of interest.

## Footnotes

1 http://www.MycoBank.org

2 http://mafft.cbrc.jp/alignment/server/index.html

3 http://purl.org/phylo/treebase/phylows/study/TB2:27599
